# Human Induced Pluripotent Stem Cells on Autologous Feeders

**DOI:** 10.1371/journal.pone.0008067

**Published:** 2009-12-02

**Authors:** Kazutoshi Takahashi, Megumi Narita, Midori Yokura, Tomoko Ichisaka, Shinya Yamanaka

**Affiliations:** 1 Center for iPS cell Research and Application, Institute for Integrated Cell-Material Sciences, Kyoto University, Kyoto, Japan; 2 Department of Stem Cell Biology, Institute for Frontier Medical Sciences, Kyoto University, Kyoto, Japan; 3 Yamanaka iPS Cell Special Project, Japan Science and Technology Agency, Kawaguchi, Japan; 4 Gladstone Institute of Cardiovascular Disease, San Francisco, California, United States of America; University of Southern California, United States of America

## Abstract

**Background:**

For therapeutic usage of induced Pluripotent Stem (iPS) cells, to accomplish xeno-free culture is critical. Previous reports have shown that human embryonic stem (ES) cells can be maintained in feeder-free condition. However, absence of feeder cells can be a hostile environment for pluripotent cells and often results in karyotype abnormalities. Instead of animal feeders, human fibroblasts can be used as feeder cells of human ES cells. However, one still has to be concerned about the existence of unidentified pathogens, such as viruses and prions in these non-autologous feeders.

**Methodology/Principal Findings:**

This report demonstrates that human induced Pluripotent Stem (iPS) cells can be established and maintained on isogenic parental feeder cells. We tested four independent human skin fibroblasts for the potential to maintain self-renewal of iPS cells. All the fibroblasts tested, as well as their conditioned medium, were capable of maintaining the undifferentiated state and normal karyotypes of iPS cells. Furthermore, human iPS cells can be generated on isogenic parental fibroblasts as feeders. These iPS cells carried on proliferation over 19 passages with undifferentiated morphologies. They expressed undifferentiated pluripotent cell markers, and could differentiate into all three germ layers via embryoid body and teratoma formation.

**Conclusions/Significance:**

These results suggest that autologous fibroblasts can be not only a source for iPS cells but also be feeder layers. Our results provide a possibility to solve the dilemma by using isogenic fibroblasts as feeder layers of iPS cells. This is an important step toward the establishment of clinical grade iPS cells.

## Introduction

Human pluripotent stem cells, both embryonic stem (ES) cells and induced Pluripotent Stem (iPS) cells, are generally maintained on mouse embryonic fibroblasts (MEF), which are mitotically inactivated by treatment with mitomycin C or γ-ray irradiation [1]–[3]. However, usage of mouse feeder cells may transfer exogenous antigens, unknown viruses, or zoonotic pathogens to iPS cells. In fact, non-human sialic acid N-glycolylneuraminic acid (Neu5Gc), which is potentially immunogenic, was detected on the surface of human ES cells maintained on MEF feeder [4]. Although feeder-free culture of human ES cells has been reported, it may lead to chromosomal instabilities of human ES cells [5], [6]. To avoid these issues, human fibroblasts from neonatal foreskin or ES cell-derived fibroblast-like were used to support self-renewal of human ES cells [7]–[11]. However, one still have to concern about existence of unidentified pathogens, such as viruses and prions in these non-autologous feeders. Since iPS cells are generated from fibroblasts, it would be ideal if the same fibroblasts can be used for the generation and maintenance of iPS cells.

## Results and Discussion

**Figure 1:**
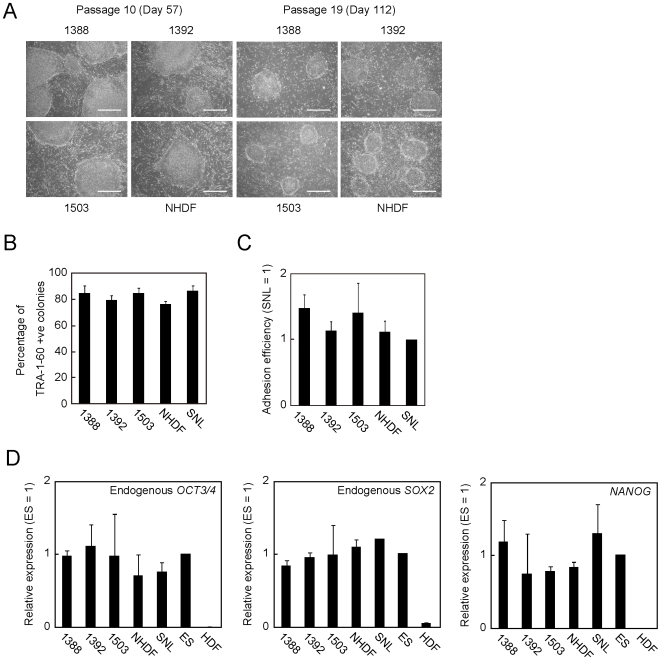
HDF can maintain self-renewal of established human iPS cells. A. Images of iPS cells maintained on each fibroblast at passage number 10 and 19. Bars indicate 200 µm. B. 201B7 iPS cells at passage number 19 were plated on each feeder, and incubated for 6 days. The graph shows the percentage of TRA-1-60 positive colonies. Three individual assays were performed. Error bars indicate standard deviation. C. The number of colonies was counted and compared with the results of SNL feeder. This graph showed the average of three independent experiments. Error bars mean standard deviation. D. RT-PCR of undifferentiated ES cell markers. 201B7 iPS cells maintained on each HDF over 100 days were lysed, and their total RNAs were purified. One microgram of RNA sample was used for cDNA synthesis. qPCR was performed with the primers for endogenous *OCT3/4*, endogenous *SOX2*, *NANOG* and *G3PDH*. Data were normalized with the value of *G3PDH*. The graphs showed the average of three experiments. Error bars indicate standard deviation.

To examine whether human fibroblasts support self-renewal of human iPS cells, we treated four independent human fibroblast lines (1388, 1392, 1503 and NHDF; see Table S1) and SNL cells [12] with mitomycin C, and seeded them on culture plates (Fig. S1). Then, we plated 201B7 iPS cell line [2] derived from 1388 fibroblasts onto these feeder cells with standard density (1∶5 dilutions). The passage number of iPS cells was 20 at this point. All the five cell lines of feeder cells were supportive for undifferentiated growth of iPS cells at least 19 additional passages (Fig. 1A). The percentage of TRA-1-60 (a marker for undifferentiated ES cells and iPS cells) positive colonies was similar among different human fibroblasts and SNL cells (Fig. 1B). No significant differences were observed in the plating efficiencies (Fig. 1C). In iPS cells at passage 2 after switching onto various HDF feeders, no significant re-activation of transgenes was observed (Fig. S2). In addition, reverse transcription polymerase chain reaction (RT-PCR) showed that the expression of ES cell marker genes such as *OCT3/4*, *SOX2* and *NANOG* were equally to those of H9 ES cells at passage 19 [1] (Fig. 1D).

Conditioned medium (CM) of MEF or SNL allows feeder-free culture of iPS cells. To test whether CM of fibroblasts could maintain self-renewal of iPS cells without feeder cells, we seeded 201B7 iPS cells onto Matrigel-coated plates in CM from each human fibroblast line or SNL. As a control, we used non-conditioned medium supplemented with bFGF. Cells in non-conditioned medium failed to form tightly packed colonies, whereas those in each CM grew healthily with typical undifferentiated ES-like morphologies (Fig. S3A). RT-PCR revealed that iPS cells maintained in each CM expressed undifferentiated ES cell marker genes such as *OCT3/4*, *SOX2*, *NANOG* and *TERT* at similar levels to those in iPS cells or human ES cells cultured on SNL feeder layers (Fig. S3B). Quantitative PCR (qPCR) confirmed that no significant alternations in the expression levels of *OCT3/4*, *SOX2* and *NANOG* transcripts among CM from different feeders (Fig. S3C). These data demonstrated that human neonatal and adult fibroblasts could be utilized as feeder cells of human iPS cells.

**Figure 2:**
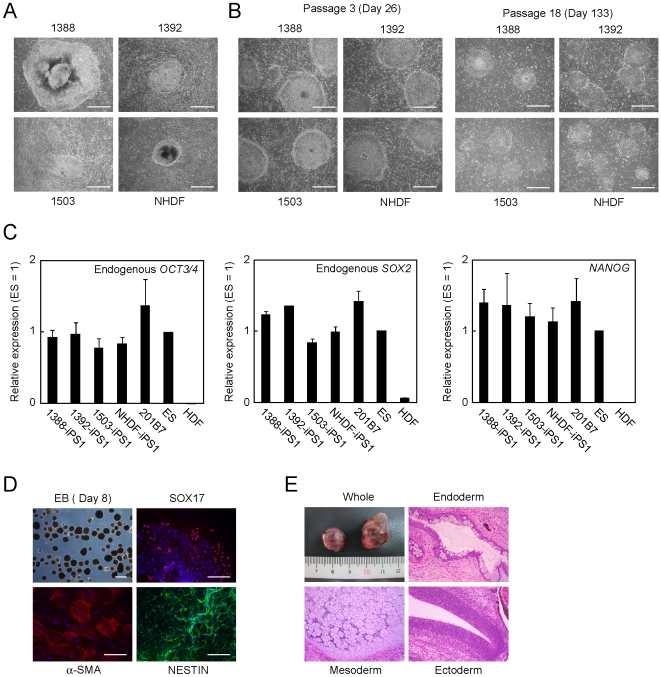
Generation and maintenance of human iPS cells on autologous feeders. A. Images of primary iPS cell colonies. We introduced 4 reprogramming factors into 1388, 1392, 1503 or NHDF. The colonies were photographed 25 days after transduction. Bars indicate 200 µm. B. Images of established iPS clones. We isolated iPS clones and transferred onto each isogenic fibroblast line. The cells at passage 3 and 18 were photographed. Bars indicate 200 µm. C. Quantification of the expression of pluripotent stem cell markers. Total RNA of iPS cells established from four independent fibroblast lines maintained on isogenic feeders, H9 ES cells and HDF was purified, and used for reverse transcription. The graphs show the average of three independent experiments. Error bars indicate standard deviation. Data were normalized with the results of *G3PDH*. D. In vitro differentiation of iPS cells. iPS cells were transferred to suspension culture to form embryoid bodies for 8 days. Embryoid bodies were transferred to gelatin-coated plated, and incubated another 8 days. The cells were stained with anti-SOX17 (red), anti-α-SMA (red) or anti-NESTIN (green) antibodies. Nucleuses were stained with Hoechst 33342 (blue). Bars indicate 100 µm. E. Teratoma formation of iPS cells. Paraffin-embedded sections were stained with hematoxylin and eosin.

Next, we examined whether human iPS cells could be established without non-autologous feeder cells. We introduced the four reprogramming factors into the four human fibroblast lines by retroviral transduction. Six days after infection, we plated the transduced cells at 5×10^5^ cells on 100-mm dishes either with SNL feeders, with isogenic human fibroblast feeder, or without feeder cells. Next day, we started cultivation using human ES cell culture medium. In the plates without feeders, the plated cells became confluent within a several days and showed an appearance resembling feeder cells. Around three weeks after transduction, ES-like colonies began to emerge on the feeder cell-like layer (Fig. 2A). We observed no significant differences in the numbers of ES-like colonies among on SNL feeders, on isogenic fibroblast feeders or feeder-free condition (Table S2). On day 25 after transduction, we picked up ES-like colonies from the plates without feeders and transferred them onto new plates with mitomycin C-treated each parental fibroblasts as feeders. Human iPS cells derived from each of the four fibroblast lines used in this study grew normally and maintained the undifferentiated morphologies on corresponding autologous feeders for at least 18 passages (Fig. 2B).

RT-PCR showed that established clones at passage number 5 expressed endogenous *OCT3/4*, *SOX2*, *NANOG* and *TERT* transcripts at similar levels to those in 201B7 iPS cells, which were established on SNL feeder cells, and H9 ES cells (Fig. S4A). The retroviruses of the four factors were effectively silenced, which is a hallmark of complete reprogramming (Fig. S4B). Even after additional 15 passages, the expression of *OCT3/4*, *SOX2* and *NANOG* in these iPS cells were comparable to those of ES cells and iPS cells maintained on SNL feeder cells (Fig. 2C). Immunoprecipitation assay with anti-methylated cytosine antibody revealed that the promoter regions of pluripotent-associated genes such as *OCT3/4* and *NANOG* locus were almost completely unmethylated in iPS cells established and maintained on the autologous feeders at passage number 5, like in H9 ES cells (Fig. S5). In addition, iPS cells generated with the autologous feeders showed normal karyotypes at least after 26 times passages (Fig. S6).

To evaluate pluripotency of iPS cells generated and maintained on autologous feeders, we performed *in vitro* differentiation assay. These iPS clones formed embryoid bodies using the floating culture condition. After 16-day differentiation, we detected SOX17 (endoderm), α-smooth muscle actin (α-SMA, mesoderm) and NESTIN (ectoderm) positive cells in the culture (Fig. 2D, Fig. S7). We also confirmed that undifferentiated markers such as *OCT3/4*, *SOX2* and *NANOG* decreased and other differentiated markers such as *AFP*, *PDGFRα* and *PAX6* increased (Fig. S8). In addition, we injected iPS cells at passage number 9 into testes of immune-deficient mice for teratoma formation. After 8 to 12 weeks, all clones we tested developed teratoma containing various tissues including gut-like epitheliums (endoderm), cartilages (mesoderm) and neural rosettes (ectoderm) (Fig. 2E). These data confirmed pluripotency of iPS cells, which were established and maintained on the autologous feeder cells.

To examine the compatibility of iPS cells and feeder cells, we plated iPS cells derived from the four HDF onto mitomycin C-treated parental fibroblasts or SNL cells with all the possible combinations. After six days, we stained the cells with TRA-1-60 antibody, and counted the number of positive colonies. More than 80% of colonies in all the combinations showed morphology of undifferentiated ES-like cells and were positive for TRA-1-60 (Fig. S9).

Our results demonstrated that human iPS cells can be generated and maintained on autologous fibroblasts as feeder layers. Furthermore, human iPS cell can be generated even without any additional feeders, since non-reprogrammed fibroblasts can serve as feeders. The maintenance of iPS cells can also be achieved without feeders, by using conditioned medium of human fibroblasts. All of the tests performed in this study revealed that iPS cells derived from four independent HDF maintained autologous feeders kept pluripotency during at least 100 day culture (Table S3). The most of reprogrammed cell colonies on isogenic feeders are uniformly undifferentiated (Fig. S9).

In general, we can obtain more than ten millions of fibroblasts from 5-mm square skin biopsy at the passage number 3 with our standard protocol [13]. For iPS cell generation with retroviruses, we need less than 1×10^5^ fibroblasts. An alternative method using an episomal vector system requires one million of fibroblasts [14]. Thus, we have enough amounts of surplus fibroblasts to be used as autologous feeder cells. We did notice that iPS cells cultured on SNL feeders are easier to passage than those on MEF and human fibroblast feeders.

Our data that all four HDF lines tested in this study could support both generation and maintenance of iPS cells, does not guarantee that every fibroblasts can be used as feeders cells for human pluripotent cells [15]. We also tested that whether 14 HDF lines were supportive for maintenance of ES cells and iPS cells. Both KhES3 ES cell line and 201B7 could grow normally on eleven out of 14 HDF lines, two MEF lines (ICR and C57BL6) and SNL [16] (Table S1, Fig. S1). In co-culture with three lines (1554, 1616 and TIG107), at least either KhES3 or 201B7 could not stay at undifferentiated state even at passage number 2. At least, among on 11 supportive fibroblasts and MEF and SNL, no significant differences were observed in growth rate of ES cells and iPS cells. Unsupportive lines are indistinguishable from HDF lines by their morphologies or growth speed. Probably, support activity for self-renewal of ES cells and iPS cells do not depend on at least passage number and donor's sex or race. Unsupportive lines tested in this study are derived from donors at 68, 77 and 81 years old. On the other hand, we found that HDF derived from donors at 69 and 73 years old could support maintenance of undifferentiated state of both ES cells and iPS cells. Further detailed analyses will be required for decision whether donor's ages of feeder cells are important or not.

Recent study by Unger and colleagues demonstrated that iPS cells derived from human fetal fibroblasts could be established and maintained on isogenic feeder cells [17]. On the other hand, we assessed that neonate and adult fibroblast-derived iPS cells can be generated and maintained on autologous feeders. Inspection of established iPS cells is always necessary before clinical trials even in autologous cell transplantation therapy. However, the culture system established in this study demonstrated that fibroblasts from an individual could play dual roles as source of iPS cells and feeder cells, probably contributing to efficient processing for clinical grade-pluripotent stem cells. Actually, the system still includes animal components such as albumin, insulin and trypsin. Xeno-free culture is basically required for therapeutic usage of iPS cells. However, even if it is for removing the xenogenic components, the culture condition should not be oppressive for pluripotent cells. Isogenic culture from the start is one of true worth of iPS cells because autologous fibroblasts can not normally inhabit when ES cells are established from blastocysts. Our result is an important step toward the generation of clinical-grade human iPS cells suitable for future medical applications.

## Materials and Methods

### Cell Culture

Human dermal fibroblasts (HDF) were purchased from Cell applications Inc or obtained from National Institute of Biomedical Innovation. HDF, 293FT and PLAT-E [18] were maintained Dulbecco's modified eagle medium (DMEM, Nacalai tesque) contained 10% fetal bovine serum (FBS, Invitrogen) and 0.5% penicillin and streptomycin (Invitrogen). The medium for human iPS cells (hES medium) consisted of DMEM/F12 (Invitrogen), 20% Knockout serum replacement (KSR, Invitrogen), 2 mM L-glutamine (Invitrogen), 1×10^−4^ M non essential amino acids (Invitrogen), 1×10^−4^ M 2-mercaptoethanol (Invitrogen) and 0.5% penicillin and streptomycin supplemented with 4 ng/ml recombinant human basic fibroblast growth factor (bFGF, WAKO).

### Generation of iPS Cells

iPS cells were established from HDF as described previously with some slight modifications [2]. In brief, we firstly introduced mouse solute carrier family 7 (cationic amino acid transporter, y+ system), member 1 (*Slc7a1*) gene which encodes ecotropic retrovirus receptor by lentiviral transduction. Transfectants were plated at 2×10^5^ cells per 60 mm dish and incubated overnight. The next day, into the cells *OCT3/4*, *SOX2*, *KLF4* and *c-MYC* were introduced by retroviral infection. Six days later, the cells were harvested by trypsinization, and plated at 5×10^5^ cells per 100 mm dish. The medium was replaced on the next day with hES cell medium, and cultured for another 20 days. At day 25 post-induction, ES-like colonies were mechanically dissociated and transferred on to 24-well plate on each isogenic feeder. We designated this point as passage 1.

### Feeder Cells

We added phosphate buffered saline (PBS, Nacalai tesque) containing 12 µg/ml mitomycin C directly into fibroblast culture in subconfluent, and incubated at 37°C for 3 hours. After treatment, the cells were washed twice with PBS and harvested by trypsinization. The cells were plated at 1×10^6^ cells per 24-well plate, 6-well plate, 3 of 60 mm dishes or 100 mm dish.

### Conditioned Medium

We plated fibroblasts at 3×10^5^ cells per 60 mm dish, and incubated overnight. Next day, the medium was replaced with 3 ml of hES medium, and incubated for 24 hours. After incubation, the supernatant of fibroblast culture was collected and filtered. We added 4 ng/ml bFGF before use.

### Differentiation

iPS cells were harvested by treatment with CTK solution consisting of 0.1 mg/ml collagenase IV (Invitrogen), 0.25% trypsin (Invitrogen), 0.1 mM CaCl_2_ (Nacalai tesque) and 20% KSR, and then suspended cell clumps in hES medium plus 10 µM Y-27632 without bFGF [19]. The cells were transferred to ultra low binding plate (Corning). After 8-day floating culture, embryoid bodies were transferred on to gelatin-coated plate, and incubated another 8 days. After incubation, the cells were fixed with PBS containing 4% paraformaldehyde and then incubated in PBS containing 5% normal goat or donkey serum (Chemicon), 1% bovine serum albumin (BSA, Nacalai tesque), and 0.2% TritonX-100. The primary antibodies were as follows; anti-SOX17 (1∶300, R & D systems), anti-α-smooth muscle actin (α-SMA, 1∶500, DAKO) and anti-NESTIN (1∶1000, Abcam). The secondary antibodies were as follows; Cyanine 3-labeled anti-goat IgG (1∶500, Zymed), Alexa 546-labeled anti-mouse IgG (1∶500, Invitrogen) and Alexa 488-labeled anti-rabbit IgG (1∶1000, Invitrogen). Nucleuses were stained with 1 µg/ml Hoechst 33342 (Invitrogen).

### Expression Analyses

We performed RT-PCR as described previously [2]. In brief, the cells were lysed with Trizol reagent (Invitrogen), and then total RNA was purified. RNA samples were treated with Turbo DNA free (Ambion) to remove genomic DNA contamination. One microgram of DNase treated RNA was used for first-strand complementary DNA (cDNA) synthesis with Rever tra ace -α- (Toyobo) and oligo dT_20_ primer. qPCR was performed using SYBR Premix ExTaq II (Takara). Primer sequences were listed in Table S4 [2], [20], [21], [22].

### Methylation Assay

Four microgram of genomic DNA was mechanically shared by sonication, and boiled at 95°C for 10 minutes. Then shared genomic DNA was incubated with pan-mouse IgG magnetic beads (Invitrogen) -conjugated anti-5-methyl cytosine antibody (Eurogentec) supplemented with 5 µg/ml BSA and 25 µg/ml yeast tRNA (Ambion) overnight at 4°C. Beads were washed three times with PBS containing 0.05% TritonX-100. Beads were suspended in 0.15 ml of TE containing 1% SDS, and incubated at 65°C for 5 minutes. The elution was repeated with an additional 0.15 ml of 1% SDS/TE. The eluates were treated with Protease K at 50°C for 2 hours, and then extracted with phenol: chloroform: isoamyl alcohol, and purified by ethanol precipitation. Primer sequences are provided in Table S4.

## Supporting Information

Figure S1 Images of mitomycin C-treated HDF, MEF and SNL. Bars indicate 200 µm.
(2.91 MB TIF)

Figure S2 The expression of four reprogramming factors in iPS cells on various HDF feeders at passage 2. RT-PCR was performed with the primers for endogenous and total (common in endogenous and transgene) OCT3/4, SOX2, KLF4 and c-MYC. Data were normalized with the value of NAT1. The graphs showed the average of triplicate. Error bars indicate standard deviation.
(0.31 MB TIF)

Figure S3 A. Image of 201B7 iPS cells maintained in CM of each HDF. Bars indicate 200 µm. B. RT-PCR of undifferentiated ES cell markers. iPS cells maintained on feeders (F) or in feeder-free culture with conditioned medium (CM) were lysed, and their total RNAs were purified. One microgram of RNA sample was used for cDNA synthesis. PCR was performed with the primers for endogenous OCT3/4, endogenous SOX2, NANOG, TERT and NAT1. C. qPCR of the expression of OCT3/4, SOX2 and NANOG in 201B7 iPS cells maintained on various feeder cells or in their conditioned medium. Data were normalized with the value of G3PDH. The graphs showed the average of three experiments. Error bars indicate standard deviation.
(1.67 MB TIF)

Figure S4 A. RT-PCR of undifferentiated ES cell markers. Total RNAs of iPS cells established from four independent fibroblast lines and maintained on each parental fibroblast were isolated and used for reverse transcription. PCR was performed with the primers for endogenous OCT3/4, endogenous SOX2, NANOG, TERT and NAT1. B. The expression of OCT3/4 (total and endogenous), SOX2 (total and endogenous) and NANOG were quantified by qPCR. Data were normalized with the value of G3PDH. The graphs showed the average of triplicate. Error bars indicate standard deviation.
(0.75 MB TIF)

Figure S5 CpG methylation statuses at promoter regions of ES cell marker genes in iPS cells maintained on autologous feeders. Immunoprecipitants by anti-5-methyl cytosine (mDIP) antibody or normal mouse IgG, or pre-immunoprecipitated DNA (Input) were used for qPCR as a template. The data was calculated as (mDIP − normal IgG)/Input. Each data was normalized by the result of H9 ES cells. The data indicate the results of qPCR in triplicate of two independent experiments. Error bars indicate standard deviation.
(0.25 MB TIF)

Figure S6 Images of G-band staining of iPS cells.
(0.68 MB TIF)

Figure S7 Images of differentiated iPS cells in vitro. iPS cells differentiated via embryoid body formation. Red or green signals indicate SOX17-, α-SMA- or NESTIN-positive cells. Nucleuses were stained with Hoechst 33342 (blue). Bars indicate 100 µm.
(4.63 MB TIF)

Figure S8 iPS cells maintained on isogenic feeders (U) or differentiated by embryoid body formation (D) were lysed with Trizol reagent. Total RNA was purified and treated with DNase to remove genomic DNA contamination. One microgram of DNase-treated RNA sample was used for first-strand cDNA synthesis with oligo dT20 primer. PCR was performed with the primers listed in Supplemental Table 2.
(0.39 MB TIF)

Figure S9 Images of iPS cells from four HDF on various HDF feeders or SNL. Red signals indicate TRA-1-60 positive cells. Nucleuses were visualized by Hoechst 33342 staining. Bars indicate 200 µm.
(3.96 MB TIF)

Table S1 The list of HDF lines used in this study.
(0.04 MB DOC)

Table S2 The number of ES-like and total colonies from four HDF on SNL, on isogenic fibroblasts, or in feeder-free condition.
(0.03 MB DOC)

Table S3 Experiments performed in this study.
(0.04 MB DOC)

Table S4 Primer sequences.
(0.06 MB DOC)
