# Supplementary figures and images for: Human Induced Pluripotent Stem Cells on Autologous Feeders
Source: PLoS One. 2009 Dec 2;4(12):e8067. doi: 10.1371/journal.pone.0008067 (PMC2780725; doi:10.1371/journal.pone.0008067)

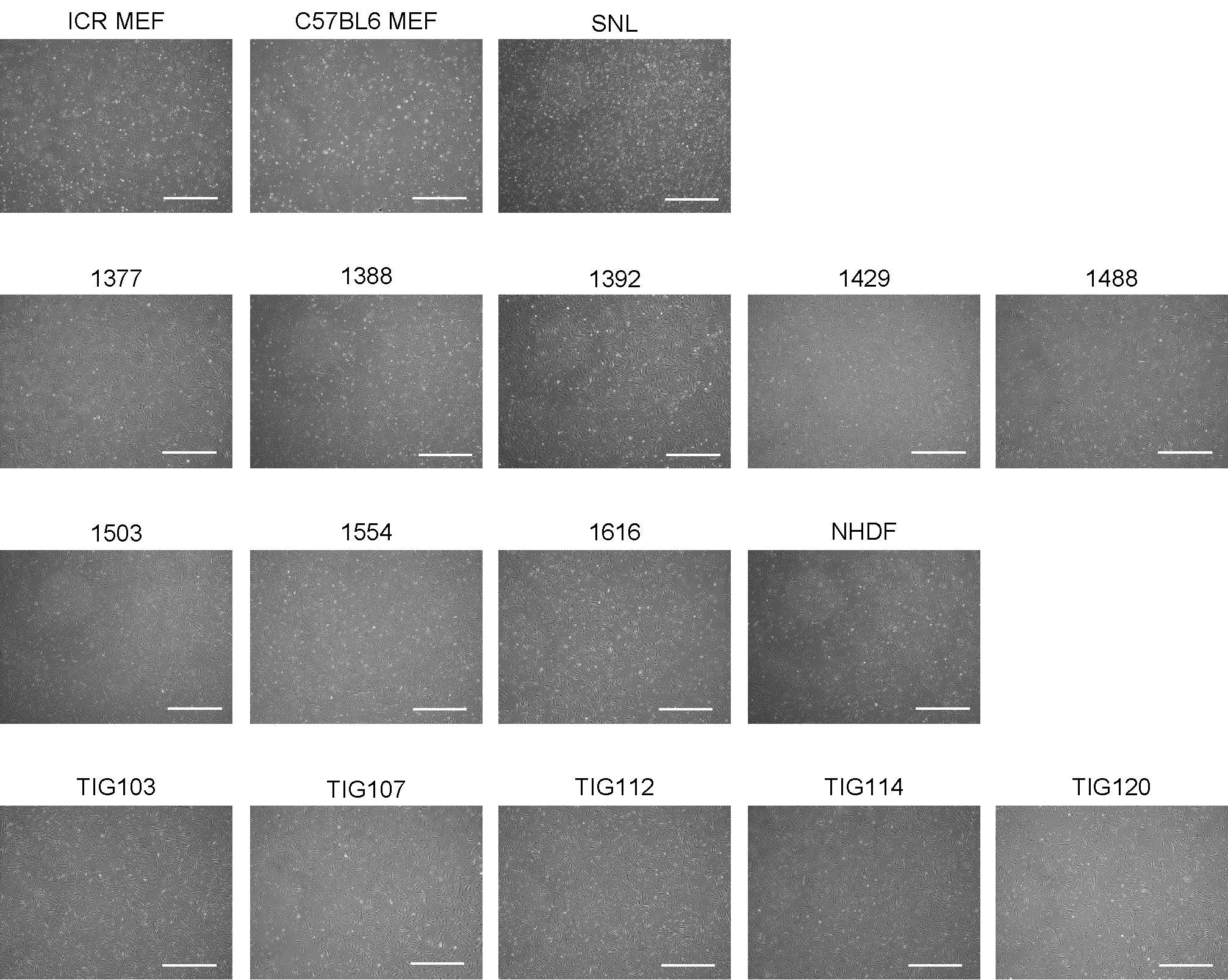

Supplement: Figure S1 — Images of mitomycin C-treated HDF, MEF and SNL. Bars indicate 200 µm. (2.91 MB TIF) [file pone.0008067.s001.tif]

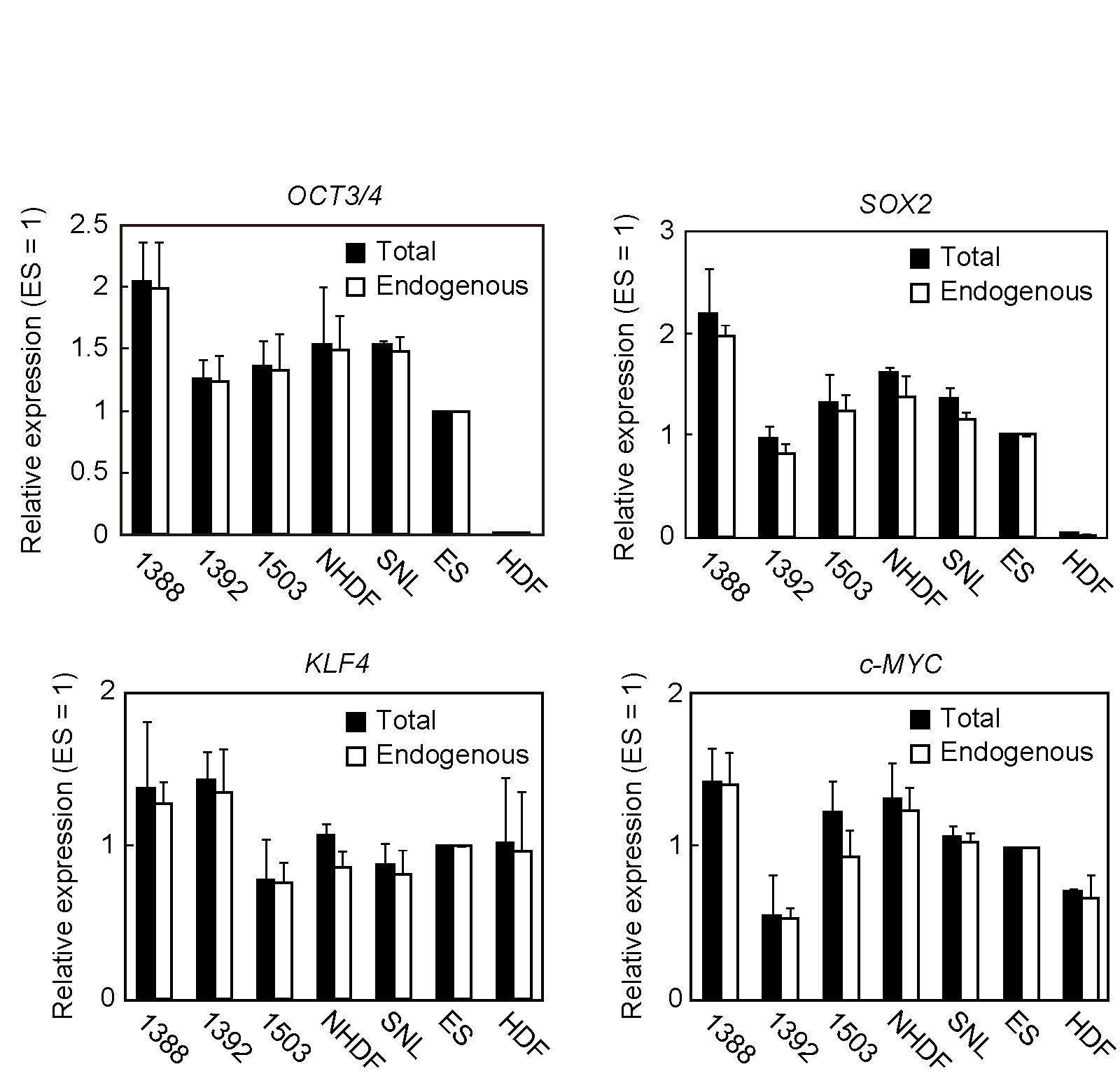

Supplement: Figure S2 — The expression of four reprogramming factors in iPS cells on various HDF feeders at passage 2. RT-PCR was performed with the primers for endogenous and total (common in endogenous and transgene) OCT3/4, SOX2, KLF4 and c-MYC. Data were normalized with the value of NAT1. The graphs showed the average of triplicate. Error bars indicate standard deviation. (0.31 MB TIF) [file pone.0008067.s002.tif]

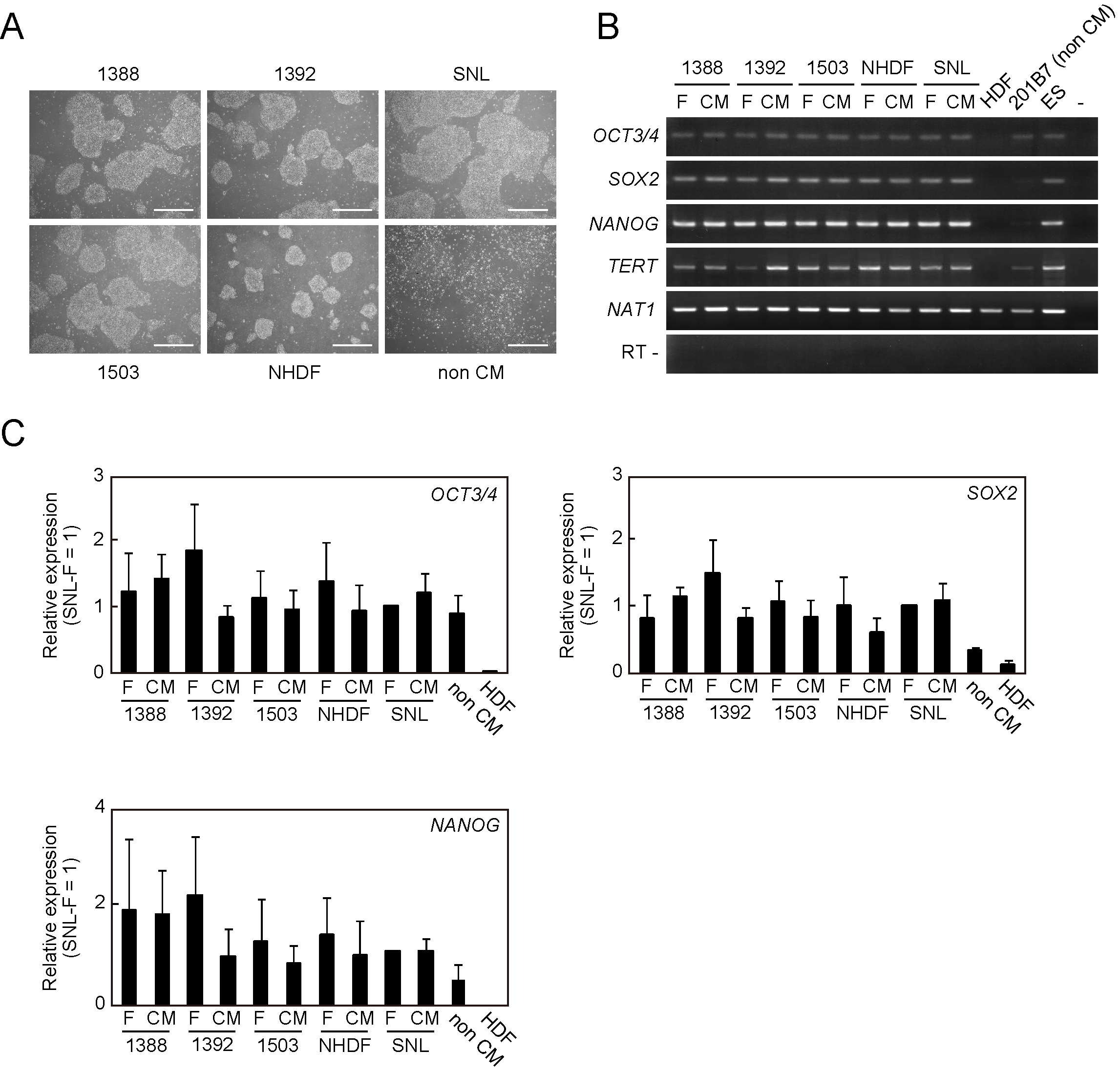

Supplement: Figure S3 — A. Image of 201B7 iPS cells maintained in CM of each HDF. Bars indicate 200 µm. B. RT-PCR of undifferentiated ES cell markers. iPS cells maintained on feeders (F) or in feeder-free culture with conditioned medium (CM) were lysed, and their total RNAs were purified. One microgram of RNA sample was used for cDNA synthesis. PCR was performed with the primers for endogenous OCT3/4, endogenous SOX2, NANOG, TERT and NAT1. C. qPCR of the expression of OCT3/4, SOX2 and NANOG in 201B7 iPS cells maintained on various feeder cells or in their conditioned medium. Data were normalized with the value of G3PDH. The graphs showed the average of three experiments. Error bars indicate standard deviation. (1.67 MB TIF) [file pone.0008067.s003.tif]

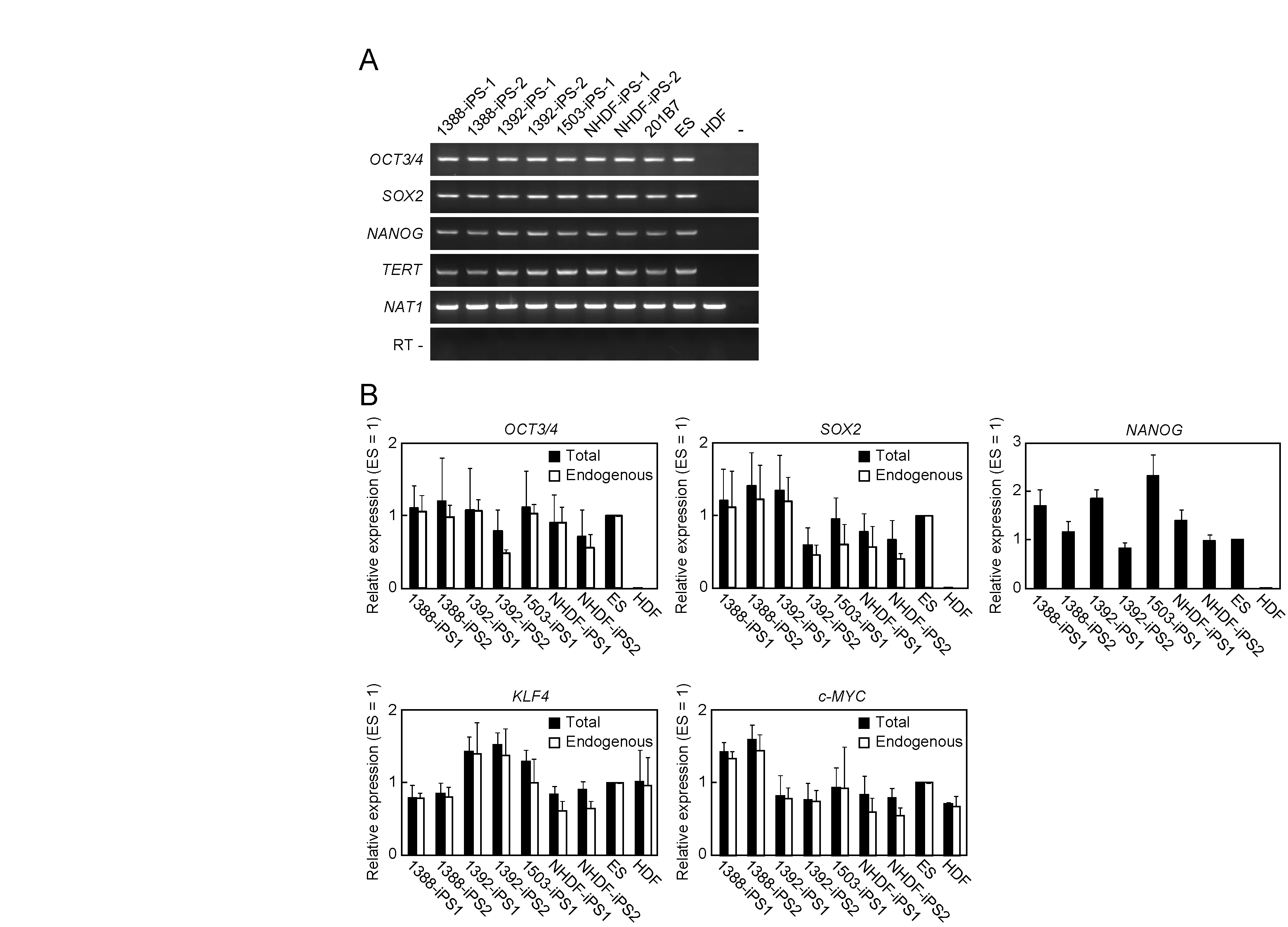

Supplement: Figure S4 — A. RT-PCR of undifferentiated ES cell markers. Total RNAs of iPS cells established from four independent fibroblast lines and maintained on each parental fibroblast were isolated and used for reverse transcription. PCR was performed with the primers for endogenous OCT3/4, endogenous SOX2, NANOG, TERT and NAT1. B. The expression of OCT3/4 (total and endogenous), SOX2 (total and endogenous) and NANOG were quantified by qPCR. Data were normalized with the value of G3PDH. The graphs showed the average of triplicate. Error bars indicate standard deviation. (0.75 MB TIF) [file pone.0008067.s004.tif]

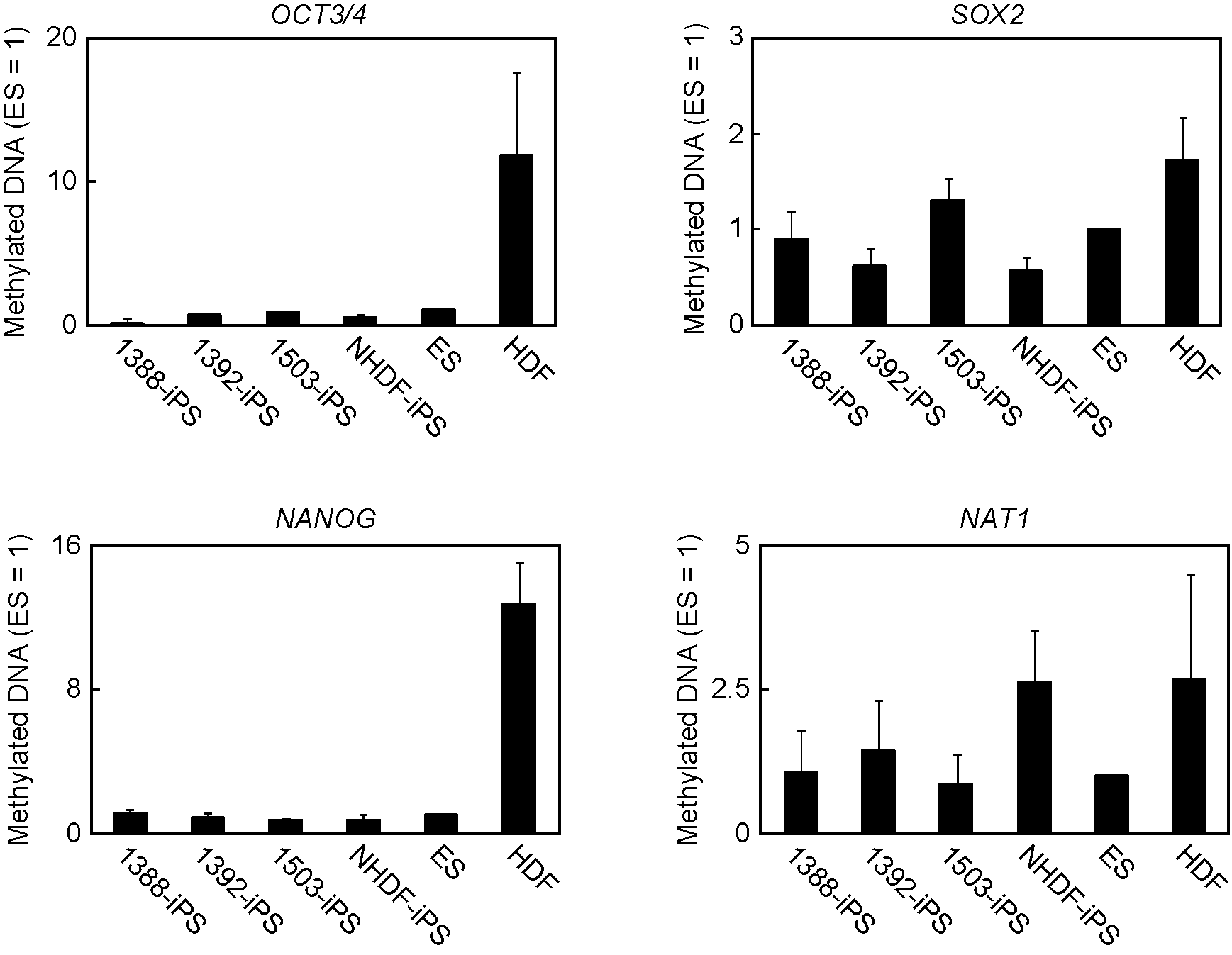

Supplement: Figure S5 — CpG methylation statuses at promoter regions of ES cell marker genes in iPS cells maintained on autologous feeders. Immunoprecipitants by anti-5-methyl cytosine (mDIP) antibody or normal mouse IgG, or pre-immunoprecipitated DNA (Input) were used for qPCR as a template. The data was calculated as (mDIP − normal IgG)/Input. Each data was normalized by the result of H9 ES cells. The data indicate the results of qPCR in triplicate of two independent experiments. Error bars indicate standard deviation. (0.25 MB TIF) [file pone.0008067.s005.tif]

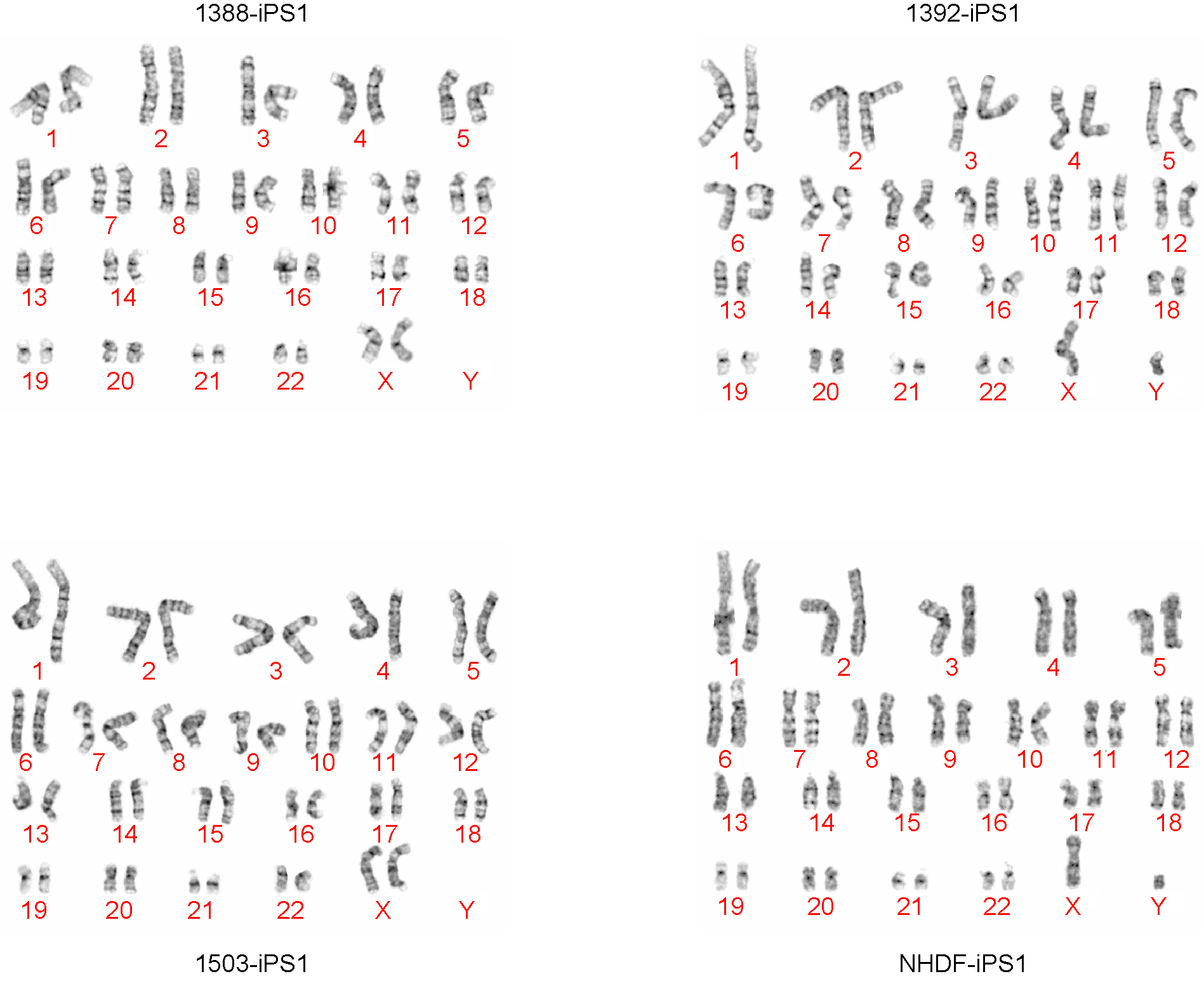

Supplement: Figure S6 — Images of G-band staining of iPS cells. (0.68 MB TIF) [file pone.0008067.s006.tif]

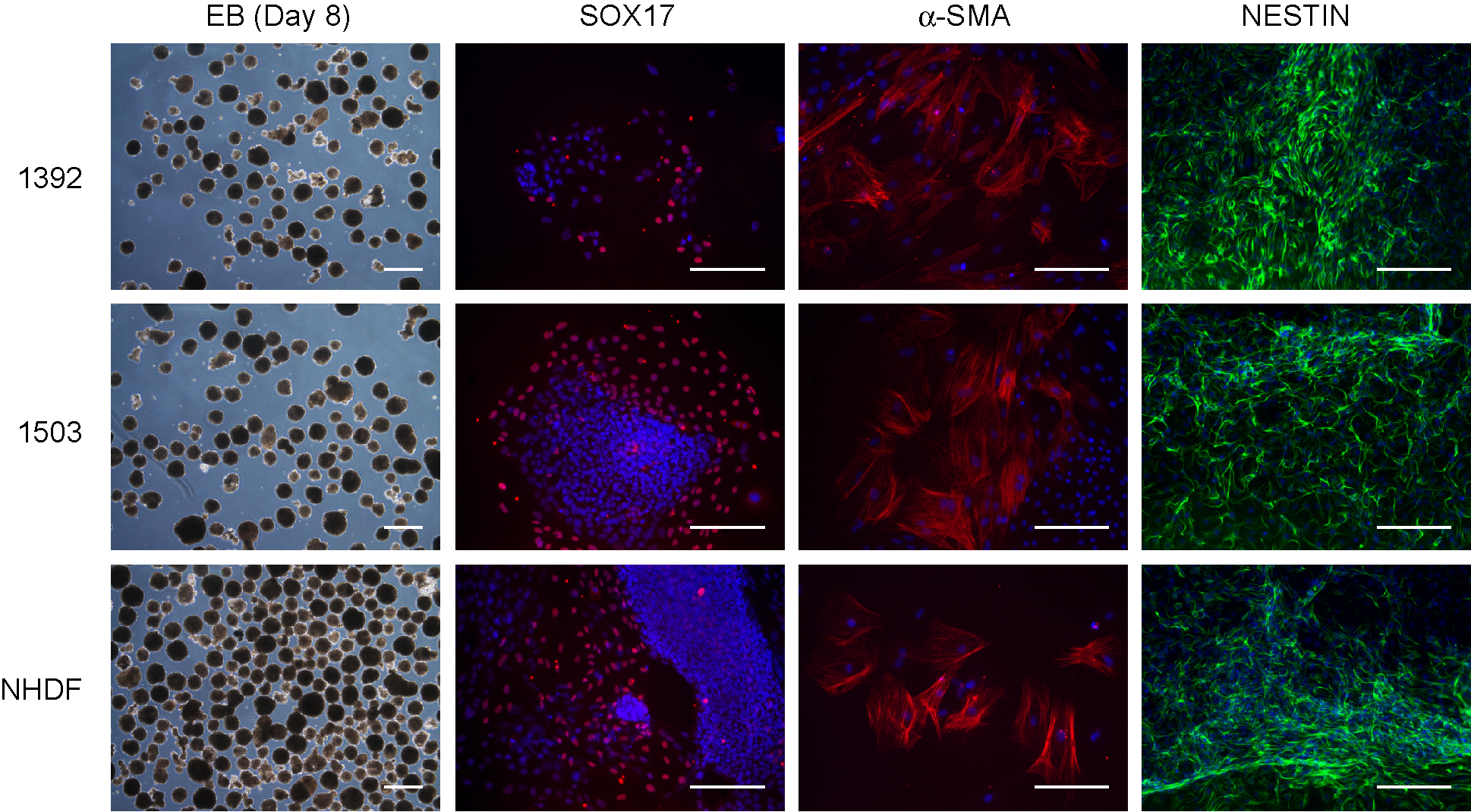

Supplement: Figure S7 — Images of differentiated iPS cells in vitro. iPS cells differentiated via embryoid body formation. Red or green signals indicate SOX17-, α-SMA- or NESTIN-positive cells. Nucleuses were stained with Hoechst 33342 (blue). Bars indicate 100 µm. (4.63 MB TIF) [file pone.0008067.s007.tif]

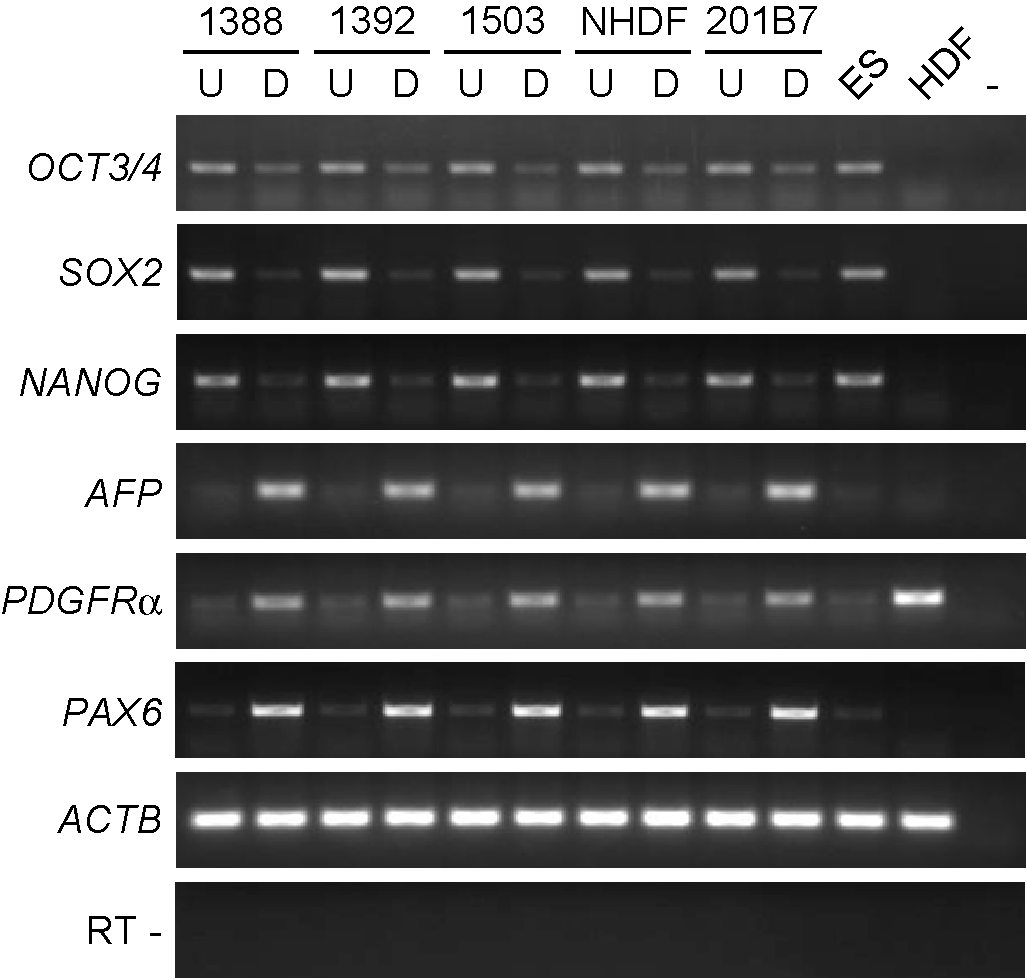

Supplement: Figure S8 — iPS cells maintained on isogenic feeders (U) or differentiated by embryoid body formation (D) were lysed with Trizol reagent. Total RNA was purified and treated with DNase to remove genomic DNA contamination. One microgram of DNase-treated RNA sample was used for first-strand cDNA synthesis with oligo dT20 primer. PCR was performed with the primers listed in Supplemental Table 2. (0.39 MB TIF) [file pone.0008067.s008.tif]

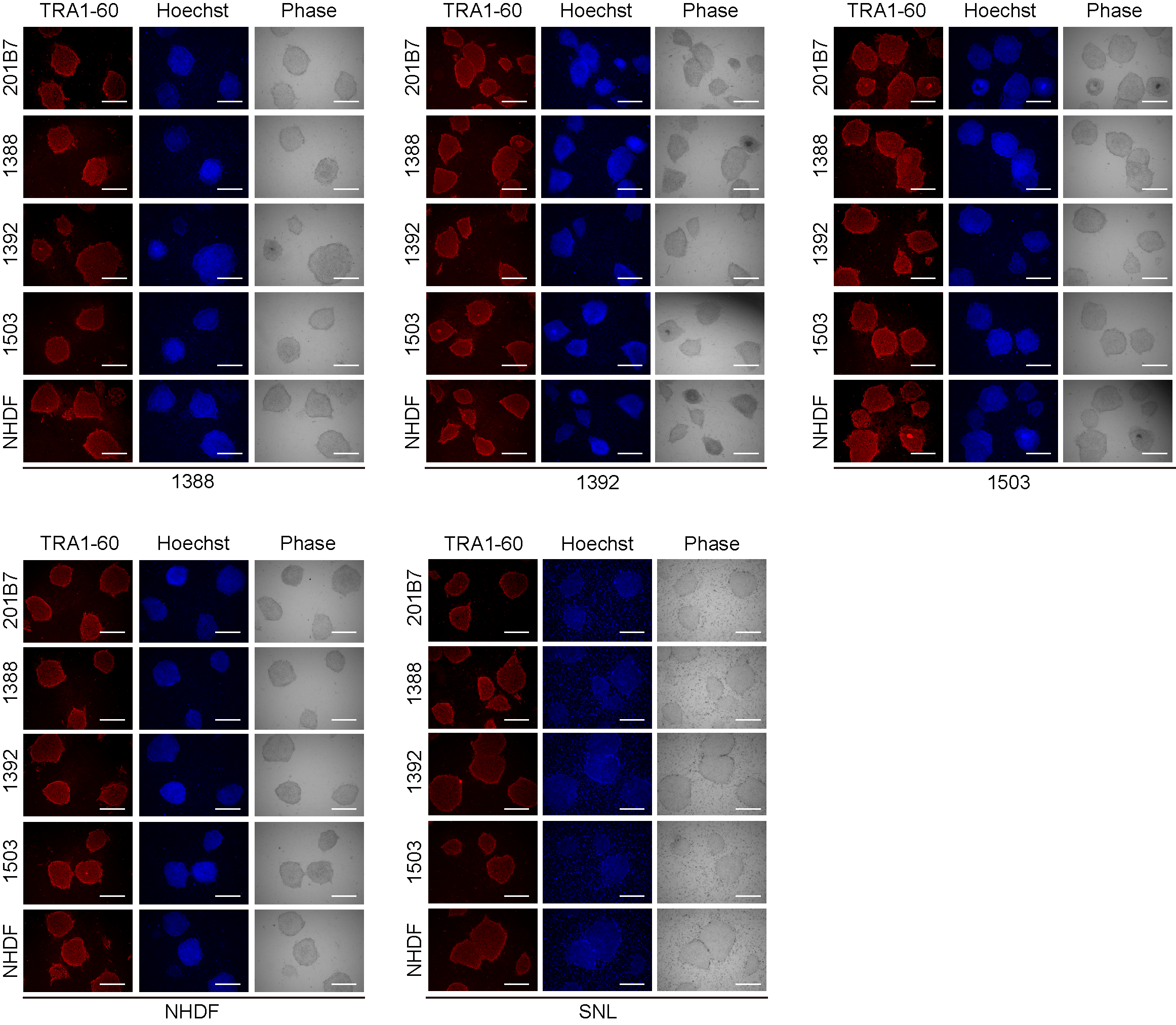

Supplement: Figure S9 — Images of iPS cells from four HDF on various HDF feeders or SNL. Red signals indicate TRA-1-60 positive cells. Nucleuses were visualized by Hoechst 33342 staining. Bars indicate 200 µm. (3.96 MB TIF) [file pone.0008067.s009.tif]
