# Supplementary material for: Human Induced Pluripotent Stem Cells on Autologous Feeders
Source: PLoS One. 2009 Dec 2;4(12):e8067. doi: 10.1371/journal.pone.0008067 (PMC2780725; doi:10.1371/journal.pone.0008067)
Supplement: Table S1 — The list of HDF lines used in this study. (0.04 MB DOC) [file pone.0008067.s010.doc]

| Cell name | Race | Sex | Age |
| --- | --- | --- | --- |
| 1377 | European descent | Female | 53 years |
| 1388 | European descent | Female | 26 years |
| 1392 | European descent | Male | 56 years |
| 1429 | European descent | Male | 45 years |
| 1488 | European descent | Female | 50 years |
| 1503 | European descent | Female | 73 years |
| 1554 | European descent | Female | 77 years |
| 1616 | Japanese | Female | 68 years |
| NHDF | European descent | Male | 0 years |
| TIG103 | Japanese | Male | 69 years |
| TIG107 | Japanese | Female | 81 years |
| TIG112 | Japanese | Female | 40 years |
| TIG114 | Japanese | Male | 36 years |
| TIG120 | Japanese | Female | 6 years |
