# Supplementary material for: Human Induced Pluripotent Stem Cells on Autologous Feeders
Source: PLoS One. 2009 Dec 2;4(12):e8067. doi: 10.1371/journal.pone.0008067 (PMC2780725; doi:10.1371/journal.pone.0008067)
Supplement: Table S2 — The number of ES-like and total colonies from four HDF on SNL, on isogenic fibroblasts, or in feeder-free condition. (0.03 MB DOC) [file pone.0008067.s011.doc]

| Source | SNL | | Isogenic | | feeder-free | |
| --- | --- | --- | --- | --- | --- | --- |
|  | ES-like | Total | ES-like | Total | ES-like | Total |
| 1388 | 5 | 79 | 4 | 62 | 2 | 3 |
| 1392 | 8 | 629 | 16 | 269 | 8 | 334 |
| 1503 | 70 | 408 | 76 | 359 | 58 | 264 |
| NHDF | 26 | 44 | 18 | 39 | 7 | 24 |
