# Supplementary material for: Human Induced Pluripotent Stem Cells on Autologous Feeders
Source: PLoS One. 2009 Dec 2;4(12):e8067. doi: 10.1371/journal.pone.0008067 (PMC2780725; doi:10.1371/journal.pone.0008067)
Supplement: Table S3 — Experiments performed in this study. (0.04 MB DOC) [file pone.0008067.s012.doc]

| Source | Clone No. | RT-PCR | EB | Teratoma | Karyotype | Methylome |
| --- | --- | --- | --- | --- | --- | --- |
| 1388 | 1 | **√** | **√** | **√** | **√** | **√** |
|  | 2 | **√** |  |  |  |  |
| 1392 | 1 | **√** | **√** | **√** | **√** | **√** |
|  | 2 | **√** |  |  |  |  |
| 1503 | 1 | **√** | **√** | **√** | **√** | **√** |
| NHDF | 1 | **√** | **√** | **√** | **√** | **√** |
|  | 2 | **√** |  |  |  |  |
