# Supplementary material for: Human Induced Pluripotent Stem Cells on Autologous Feeders
Source: PLoS One. 2009 Dec 2;4(12):e8067. doi: 10.1371/journal.pone.0008067 (PMC2780725; doi:10.1371/journal.pone.0008067)
Supplement: Table S4 — Primer sequences. (0.06 MB DOC) [file pone.0008067.s013.doc]

| Gene | Sequence (5' to 3') | Reference |
| --- | --- | --- |
| *OCT3/4* (Endogenous) | GAC AGG GGG AGG GGA GGA GCT AGG | 2 |
|  | CTT CCC TCC AAC CAG TTG CCC CAA AC |  |
| *OCT3/4* (Total) | CCC TGG TGC CGT GAA GCT GGA GAA GG | 2 |
|  | TAC TGG TTC GCT TTC TCT TTC GGG CCT G |  |
| *SOX2* (Endogenous) | GGG AAA TGG GAG GGG TGC AAA AGA GG | 2 |
|  | TTG CGT GAG TGT GGA TGG GAT TGG TG |  |
| *SOX2* (Total) | ACG ACG TGA GCG CCC TGC AGT ACA A | 2 |
|  | GCT GGA GCT GGC CTC GGA CTT GAC C |  |
| *NANOG* | TCT CTC CTC TTC CTT CCT CCA TG | 2 |
|  | CTG TTT GTA GCT GAG GTT CAG GAT G |  |
| *TERT* | CCT GCT CAA GCT GAC TCG ACA CCG TG | 2 |
|  | GGA AAA GCT GGC CCT GGG GTG GAG C |  |
| *KLF4* (Endogenous) | GAT TAC GCG GGC TGC GGC AAA ACC TAC ACA | This study |
|  | TGA TTG TAG TGC TTT CTG GCT GGG CTC C |  |
| *KLF4* (Total) | CAT GCC AGA GGA GCC CAA GCC AAA GAG GGG | This study |
|  | CGC AGG TGT GCC TTG AGA TGG GAA CTC TTT |  |
| *c-MYC* (Endogenous) | TTT CTG AAG AGG ACT TGT TGC GGA AAC GAC | This study |
|  | TCA GCC AAG GTT GTG AGG TTG CAT TTG ATC |  |
| *c-MYC* (Total) | GCC GCC GCC TCA GAG TGC ATC GAC | This study |
|  | CGA GTG GAG GGA GGC GCT GCG TAG |  |
| *AFP* | AAA TGC GTT TCT CGT TGC TT | 20 |
|  | GCC ACA GGC CAA TAG TTT GT |  |
| *PDGFRα* | ACA GGT TGG TGT GGG TTC AT | 20 |
|  | CTG CAT CTT CCA AAG CAT CA |  |
| *PAX6* | ACC CAT TAT CCA GAT GTG TTT GCC CGA G | 2 |
|  | ATG GTG AAG CTG GGC ATA GGC GGC AG |  |
| *NAT1* | ATT CTT CGT TGT CAA GCC GCC AAA GTG GAG | 21 |
|  | AGT TGT TTG CTG CGG AGT TGT CAT CTC GTC |  |
| *G3PDH* | ACC ACA GTC CAT GCC ATC AC | 22 |
|  | TCC ACC ACC CTG TTG CTG TA |  |
| *ACTB* | CAA TGT GGC CGA GGA CTT TG | 20 |
|  | CAT TCT CCT TAG AGA GAA GTG G |  |
| OCT3/4-ChIP | TTG CCA GCC ATT ATC ATT CA | 2 |
|  | TAT AGA GCT GCT GCG GGA TT |  |
| SOX2-ChIP | GAG AAG GGC GTG AGA GAG TG | 2 |
|  | AAA CAG CCA GTG CAG GAG TT |  |
| NANOG-ChIP | GAT TTG TGG GCC TGA AGA AA | 2 |
|  | GGA AAA AGG GGT TTC CAG AG |  |
| NAT1-ChIP | AGG GTT CGG GGG AGG TAA GGG TGC | 2 |
|  | AGG GTT GCG TGC GTA AAG CCG GAG |  |
